# A modified truncated distribution for modeling the heavy tail, engineering and environmental sciences data

**DOI:** 10.1371/journal.pone.0249001

**Published:** 2021-04-06

**Authors:** Ahtasham Gul, Muhammad Mohsin, Muhammad Adil, Mansoor Ali

**Affiliations:** 1 Department of Statistics, COMSATS University Islamabad, Lahore Campus, Lahore, Pakistan; 2 Pakistan Bureau of Statistics, Islamabad, Pakistan; 3 Ministry of PD&SI, Islamabad, Pakistan; Tongii University, CHINA

## Abstract

Truncated models are imperative to efficiently analyze the finite data that we observe in almost all the real life situations. In this paper, a new truncated distribution having four parameters named Weibull-Truncated Exponential Distribution (W-TEXPD) is developed. The proposed model can be used as an alternative to the Exponential, standard Weibull and shifted Gamma-Weibull and three parameter Weibull distributions. The statistical characteristics including cumulative distribution function, hazard function, cumulative hazard function, central moments, skewness, kurtosis, percentile and entropy of the proposed model are derived. The maximum likelihood estimation method is employed to evaluate the unknown parameters of the W-TEXPD. A simulation study is also carried out to assess the performance of the model parameters. The proposed probability distribution is fitted on five data sets from different fields to demonstrate its vast application. A comparison of the proposed model with some extant models is given to justify the performance of the W-TEXPD.

## 1 Introduction

Truncated probability models are efficiently used when stochastic variable is confined in some domains. They are required in almost every field like astronomy, epidemiology, biometry, engineering and economy. For instance, the government is interested to know the population of families who are living in New York city having monthly income more than 50,000 US dollars. Another example is recruitment of police officials who meet the minimum prerequisite academic qualification. In engineering, the measurements are taken by using a detector which detects the signals above a specific limit and the weak signals are not taken into account. In all the above situations we need truncated probability distributions to model them.

Weibull and Exponential distributions are immensely utilized in reliability and lifetime analysis due to their simplicity and easy mathematical manipulations. The Weibull distribution is generated by the Swedish physicist [1]. It is commonly used for modeling reliability, electrical engineering [2], mechanical engineering [3], life time and environmental sciences data due to wide-variety of shapes. A considerable literature discussing the methods of estimation of Weibull parameters is given by [4, 5]. [6] stating that Weibull distribution becomes reversed J-shaped, exponential and bell shaped for the shape parameter <, = and > 0 respectively. A comprehensive account on truncated Weibull distribution is given by [7, 8]. [9] fits the truncated Weibull distribution in different areas like to analyse the diameter of trees by truncating data at a specific threshold level and to infer the height of small trees. [10] studies the method of moments to compute the moment expression for two parameters, three-parameters and truncated (left, right and doubly) Weibull distributions. Exponential distribution is also famous for modeling the data due to availability of good estimators, and its nice mathematical properties (e.g. being memory less). [11] defines the maximum likelihood estimator of scale parameter for Exponential distribution.

[12] computes the parameter estimates of truncated Gamma probability density function (pdf). [13] distinguishes the worth of truncated probability density function in hydrology by computing the truncated moment expressions (TMEs) as well as complete moments of different densities and notify that complete moments are regarded as a special case of truncated moment expressions. [14] utilizes both skew-Cauchy (SK-CD) and truncated skew-Cauchy (TSK-CD) probability functions for modeling the exchange rate between the U.K pound sterling and the U.S dollar from 1800 to 2003 and verdicts that TSK-CD is a better probability function to model the data set contrary to SK-CD. [15] studies the truncated version of the Birnbaum-Saunders (BS) distribution to enhance a forecasting of actuarial model, specifically for modeling data regarding insurance payments that establish a deduction.

In the field of hydrology, [16] uses the generalized exponential (GE) distribution to study the flood frequency for Polish Rivers. [17] employs Weibull density function to study accrual failure detector and calls it “Weibull Distribution Failure Detector for Cloud Computing”. [18] introduces a new generalized form of Weibull probability model i.e. “Alpha logarithmic transformed Weibull distribution” (ALTW) to model the failure time of turbocharger of engine.

In engineering, [19] fitted the micro-level spatial joint and macro-level model with conditional autoregressive (CAR) to analyze the zonal crash using three years urban highway data in the USA. They conclude that micro-level model better fits the data. [20] proposes a new Bayesian Spatio-temporal model to study the association between frequency of free way incidence and other risk factors. [21] considers the mixed logit model to identify the main factors of single and multiple vehicle accidents. Similarly, in another study [22] applies the mixed logit model to analyze the significant factors of single vehicle (SV) and multiple vehicle (MV) accidents by using the 10 years truck drivers data at rural highway of the USA. [23] develops Weibull-Lindely distribution by compounding of two distributions and highlights its worth by fitting it on three medica data sets. [24] introduces U-statistics for Weibull distribution parameters and compares it with nine parameter estimation techniques.

Some distinct characteristics motivated us to demonstrate the W-TEXPD like: (i) it is distinctive by the induction of a new scale parameter obtained from the new truncated transformed distribution along with the usual induction of location parameter; (ii) the W-TEXPD shows monotonic, non-monotonic and bathtub shaped hazard rates which make the W-TEXPD a better model than those lifetime models that only demonstrate constant or monotonically increasing/decreasing hazard rates; (iii) it can be viewed that various known lifetime classical models are the special cases of W-TEXPD; (iv) it can be observed that W-TEXPD is appropriate for fitting the scattered, skewed (spread) and/or heavy tailed (flat curved) data which may not be appropriately fitted by other typical probability density functions; and (v) the results achieved by Monte Carlo simulation study for different sample sizes reveals the stability of the model parameters. Finally, we intend to find that how well W-TEXPD performs as compared to several renowned classical lifetime models by using five data sets having skewed and heavy tailed data.

The manuscript is sorted as: In Section 2, W-TEXPD is described and its characteristics such as hazard function, cumulative hazard function, raw and central moments, skewness, kurtosis, Shannon’s entropy and order statistics are derived. In Section 3, Maximum likelihood estimates of the model parameters are obtained. In Section 4, Monte Carlo simulation study is performed to examine the performance of W-TEXPD for different choices of the model parameters. In Section 5, the feasibility of the proposed model is studied by fitting it to the real data sets and comparing with some baseline models. Some concluding remarks are recorded in Section 6.

## 2 Weibull-Truncated Exponential distribution (W-TEXPD)

[25] suggests a new method for generating a family of truncated distributions called T-*X*_*T*_ family of distributions by using a new function given as

$$\begin{array}{c} W\left(F\left(x_{T}\right)\right)=-\log\left\{1-F\left(x|x>\tau \right)\right\}. \end{array}$$ (1)

Let X be a non-negative random variable truncated on left having probability density function (pdf) *f*(*x*_*T*_) and distribution function (cdf) *F*(*x*_*T*_) on domain [*τ*, ∞). Also let T be a random variable with pdf r(t) and cdf R(t) on interval [−∞ ≤ *a* ≤ *t* ≤ *b* ≤ ∞).

Then the cdf of T-*X*_*T*_ family of distributions is

$$\begin{array}{c} G\left(x_{T}\right)=\int_{\tau }^{-\log\{1-F(x|x>\tau )\}}r(t)dt, \end{array}$$ (2)

$$\begin{array}{c} G\left(x_{T}\right)=R[-\log\{1-F(x_{T})\}], \end{array}$$ (3)

where R(t) is the cdf of random variable T, while the corresponding pdf of T-*X*_*T*_ family of distributions is

$$\begin{array}{c} g\left(x_{T}\right)=r[-\log\{1-F(x|x>\tau )\;\}]\frac{f(x|x>\tau )\;}{1-F(x|x>\tau )\;},\;x>\tau . \end{array}$$ (4)

$$\begin{array}{c} g\left(x_{T}\right)=r[-\log\{1-F(x_{T})\}]\{\frac{f(x_{T})}{1-F(x_{T})}\}, \end{array}$$ (5)

$$\begin{array}{c} g\left(x_{T}\right)=r[H(x_{T})]h\left[x_{T}\right]. \end{array}$$ (6)

The idea presented in Eq (2) is extended by the method of generating a new family of distributions called T-X family of distributions proposed by [26] which is the extension of Beta Generated distributions originally introduced by [27].

Suppose X be an exponential random variable having density function

$$\begin{array}{c} f\left(x\right)=\theta e^{-\theta x}, \end{array}$$ (7)

with corresponding cdf

$$\begin{array}{c} F\left(x\right)=1-\theta e^{-\theta x}. \end{array}$$ (8)

The T-Truncated Exponential distribution defined by [25] is expressed as:

$$\begin{array}{c} g(x)=\theta r\{\theta (x-a)\}. \end{array}$$ (9)

Let T be the Weibull random variable having pdf

$$\begin{array}{c} r\left(t\right)=\frac{\beta }{\alpha^{\beta }}t^{\beta -1}e^{-{\left(t/\alpha \;\right)}^{\beta }},\;0<t<\infty . \end{array}$$ (10)

The Weibull-Truncated exponential distribution (W-TEXPD) is defined by using Eq (9) as

$$\begin{array}{c} g\left(x\right)={\left(\frac{\theta }{\alpha }\right)}^{\beta }\beta {\left(x-\tau \right)}^{\beta -1}e^{-{\left\{\frac{\theta (x-\tau )}{\alpha }\right\}}^{\beta }}\;\tau <x<\infty . \end{array}$$ (11)

Where

*τ* ————> Location parameter.

*θ*, *α*———–> Scale parameter.

*β* ————> Shape parameter.

**Some special cases of W-TEXPD**

- W-TEXPD reduces to Exponential distribution for *τ* = 0 and *θ*, *β* = 1.
- W-TEXPD reduces to Weibull for *τ* = 0, *θ* = 1.
- W-TEXPD reduces to Shifted Gamma-Weibull [28] and three parameter Weibull [29] distribution for *θ* = 1.

**Fig 1:**
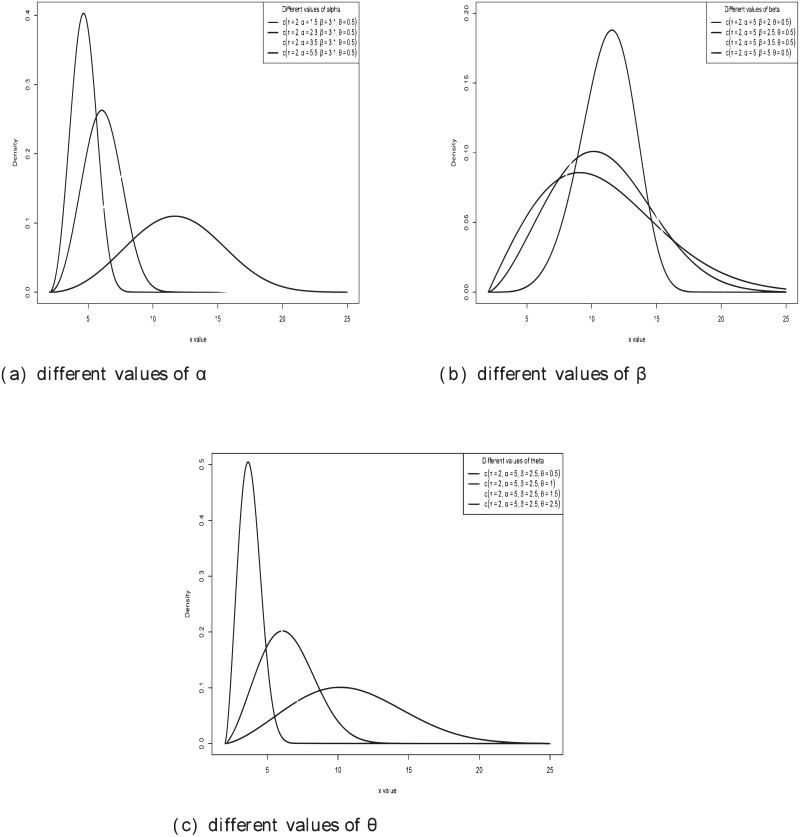
PDF of W-TEXPD for different values of *α*, *β* and *θ*.

Fig 1 displays different shapes of W-TEXPD for different values of the parameters.

**Some characteristics of W-TEXPD are**:

**Lemma 2.0.1**. *The hazard function of W-TEXPD is*

$$\begin{array}{c} h\left(t\right)=\frac{f(t)}{1-F(t)}=\frac{f(t)}{R(t)}.\\ h\left(t\right)=\beta {\left(\frac{\theta }{\alpha }\right)}^{\beta }{\left(t-\tau \right)}^{\beta -1}. \end{array}$$ (12)

**Fig 2:**
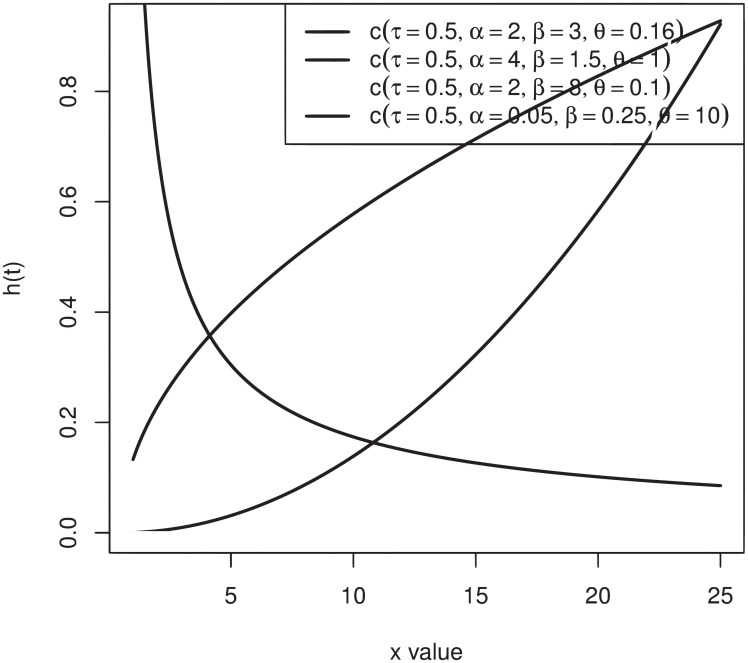
h(t) of W-TEXPD for different values of *α*, *β* and *θ*.

Fig 2 highlights that the W-TEXPD can model both monotonically and non-monotonically hazard rate shapes with different values of the parameters.

**Lemma 2.0.2**. *The cumulative hazard function of W-TEXPD is computed as*

$$\begin{array}{c} H\left(t\right)=\int_{0}^{x}h(t)dt.\\ H\left(t\right)={\left(\frac{\theta }{\alpha }\right)}^{\beta }{\left(x-\tau \right)}^{\beta },\;\beta >0. \end{array}$$ (13)

**Lemma 2.0.3**. *The p^th^ percentile of W-TEXPD is given by*

$$\begin{array}{c} G(x)=P.\\ 1-e^{-{\left\{\frac{\theta (x-\tau )}{\alpha }\right\}}^{\beta }}=P,\\ x=\tau +\frac{\alpha }{\theta }\log\left(1-P\right). \end{array}$$ (14)

**Lemma 2.0.4**. *The first four raw moments of W-TEXPD are given by*

$$\begin{array}{c} E\left(X\right)=\int_{\tau }^{\infty }xg(x)dx,\\ E\left(X\right)=\tau +\frac{\alpha }{\theta }\Gamma \left(\frac{1}{\beta }+1\right). \end{array}$$ (15)

$$\begin{array}{c} E\left(X^{2}\right)=\beta {\left(\frac{\theta }{\alpha }\right)}^{\beta }\int_{\tau }^{\infty }x^{2}{\left(x-\tau \right)}^{\beta -1}e^{-{\left\{\frac{\theta (x-\tau )}{\alpha }\right\}}^{\beta }}dx,\\ E\left(X^{2}\right)=\tau^{2}+{\left(\frac{\alpha }{\theta }\right)}^{2}\Gamma \left(\frac{2}{\beta }+1\right)+2\frac{\tau \alpha }{\theta }\Gamma \left(\frac{1}{\beta }+1\right). \end{array}$$ (16)

$$\begin{array}{c} E\left(X^{3}\right)=\int_{a}^{\infty }x^{3}g\left(x\right)dx,\\ E\left(X^{3}\right)=\tau^{3}+{\left(\frac{\alpha }{\theta }\right)}^{3}\Gamma \left(\frac{3}{\beta }+1\right)+3\tau {\left(\frac{\alpha }{\theta }\right)}^{2}\Gamma \left(\frac{2}{\beta }+1\right)+3\tau^{2}(\frac{\alpha }{\theta })\Gamma \left(\frac{1}{\beta }+1\right). \end{array}$$ (17)

$$\begin{array}{c} E\left(X^{4}\right)=\tau^{4}+4\tau^{3}(\frac{\alpha }{\theta })\Gamma \left(\frac{1}{\beta }+1\right)+6\tau^{2}{\left(\frac{\alpha }{\theta }\right)}^{2}\Gamma \left(\frac{2}{\beta }+1\right)+4\tau {\left(\frac{\alpha }{\theta }\right)}^{3}\Gamma \left(\frac{3}{\beta }+1\right)+{\left(\frac{\alpha }{\theta }\right)}^{4}\Gamma \left(\frac{4}{\beta }+1\right). \end{array}$$ (18)

**Lemma 2.0.5**. *The first four central moments of W-TEXPD are given by*

$$\begin{array}{c} \mu_{1}=0. \end{array}$$ (19)

$$\begin{array}{c} \mu_{2}=Var\left(x\right)=E\left(x^{2}\right)-{\left\{E(x)\right\}}^{2},\\ Var\left(x\right)={\left(\frac{\alpha }{\theta }\right)}^{2}[\Gamma \left(\frac{2}{\beta }+1\right)-{\left\{\Gamma (\frac{1}{\beta }+1)\right\}}^{2}]. \end{array}$$ (20)

$$\begin{array}{c} \mu_{3}={\mu_{3}}^{/}-3{\mu_{1}}^{/}{\mu_{2}}^{/}+2{\left({\mu_{1}}^{/}\right)}^{3},\\ \mu_{3}={\left(\frac{\alpha }{\theta }\right)}^{3}\Gamma \left(\frac{3}{\beta }+1\right)+\Gamma \left(\frac{1}{\beta }+1\right)[6\tau^{2}(\frac{\theta }{\alpha }-\frac{\alpha }{\theta })-3\tau^{2}(\frac{\alpha }{\theta })+2{\left(\frac{\theta }{\alpha }\right)}^{2}+6\tau {\left(\frac{\theta }{\alpha }\right)}^{2}]. \end{array}$$ (21)

$$\begin{array}{c} \mu_{4}={\mu_{4}}^{/}-4{\mu_{1}}^{/}{\mu_{3}}^{/}+6{\left({\mu_{1}}^{/}\right)}^{2}{\mu_{2}}^{/}-3{\left({\mu_{1}}^{/}\right)}^{4},\\ \mu_{4}=[\begin{array}{c} 4\tau^{3}(\frac{\theta }{\alpha })\Gamma \left(\frac{1}{\beta }+1\right)-6\tau^{2}{\left(\frac{\theta }{\alpha }\right)}^{2}\Gamma \left(\frac{2}{\beta }+1\right)-12\tau^{2}(\frac{\theta }{\alpha })\Gamma \left(\frac{1}{\beta }+1\right)-4\tau \Gamma \left(\frac{3}{\beta }+1\right)\\ +12\tau {\left(\frac{\alpha }{\theta }\right)}^{2}{\left\{\Gamma (\frac{1}{\beta }+1)\right\}}^{3}\\ -6\tau^{2}{\left(\frac{\alpha }{\theta }\right)}^{2}\{\Gamma \left(\frac{2}{\beta }+1\right)+{\left\{\Gamma (\frac{1}{\beta }+1)\right\}}^{2}-{\left\{\Gamma (\frac{1}{\beta }+1)\right\}}^{2}\Gamma \left(\frac{2}{\beta }+1\right)\}\\ +8\tau^{3}(\frac{\alpha }{\theta })\Gamma \left(\frac{1}{\beta }+1\right)-{\left(\frac{\alpha }{\theta }\right)}^{4}\Gamma \left(\frac{1}{\beta }+1\right)[4\Gamma \left(\frac{3}{\beta }+1\right)+3{\left\{\Gamma (\frac{1}{\beta }+1)\right\}}^{3}] \end{array}]. \end{array}$$ (22)

**Lemma 2.0.6**. *The skewness and kurtosis of W-TEXPD are defined as*

$$\begin{array}{c} Skewness=\frac{\mu_{3}}{{\left(\mu_{2}\right)}^{3/2\;}},\\ Skewness=\frac{{\left(\frac{\alpha }{\theta }\right)}^{3}\Gamma \left(\frac{3}{\beta }+1\right)+\Gamma \left(\frac{1}{\beta }+1\right)[6\tau^{2}(\frac{\theta }{\alpha }-\frac{\alpha }{\theta })-3\tau^{2}(\frac{\alpha }{\theta })+2{\left(\frac{\theta }{\alpha }\right)}^{2}+6\tau {\left(\frac{\theta }{\alpha }\right)}^{2}]}{{\left(\frac{\alpha }{\theta }\right)}^{3}{\left[\Gamma \left(\frac{2}{\beta }+1\right)-{\left\{\Gamma (\frac{1}{\beta }+1)\right\}}^{2}\right]}^{{}^{3/2\;}}}. \end{array}$$ (23)

$$\begin{array}{c} Kurtosis=\frac{\mu_{4}}{{\left(\mu_{2}\right)}^{2}},\\ Kurtosis=\frac{\left[\begin{array}{c} 4\tau^{3}(\frac{\theta }{\alpha })\Gamma \left(\frac{1}{\beta }+1\right)-6\tau^{2}{\left(\frac{\theta }{\alpha }\right)}^{2}\Gamma \left(\frac{2}{\beta }+1\right)-12\tau^{2}(\frac{\theta }{\alpha })\Gamma \left(\frac{1}{\beta }+1\right)\\ -4\tau \Gamma \left(\frac{3}{\beta }+1\right)+12\tau {\left(\frac{\alpha }{\theta }\right)}^{2}{\left\{\Gamma (\frac{1}{\beta }+1)\right\}}^{3}-6\tau^{2}{\left(\frac{\alpha }{\theta }\right)}^{2}\Gamma \left(\frac{2}{\beta }+1\right)\\ +{\left\{\Gamma (\frac{1}{\beta }+1)\right\}}^{2}-{\left\{\Gamma (\frac{1}{\beta }+1)\right\}}^{2}\Gamma \left(\frac{2}{\beta }+1\right)+8\tau^{3}(\frac{\alpha }{\theta })\Gamma \left(\frac{1}{\beta }+1\right) \end{array}\right]}{{\left(\frac{\alpha }{\theta }\right)}^{4}{\left[\Gamma \left(\frac{2}{\beta }+1\right)-{\left\{\Gamma (\frac{1}{\beta }+1)\right\}}^{2}\right]}^{2}}. \end{array}$$ (24)

**Theorem 2.1**. *Let* *X*_*T*_ *be a stochastic variable following W-TEXPD, then Shannon entropy is given by*

$$\begin{array}{c} \eta_{x_{T}}=1+\frac{\left(\beta -1\right)}{\beta }\{C+\beta \ln(\frac{\theta }{\alpha })\}-\ln\{\beta {\left(\frac{\theta }{\alpha }\right)}^{\beta }\}. \end{array}$$

*Proof*. The Shannon entropy of a random variable *X*_*T*_ is a measure of uncertainty given as

$$\begin{array}{c} \eta_{x_{T}}=E\left\{\ln g\left(x_{T}\right)\right\}=\int_{\tau }^{\infty }\left\{-\ln g\left(x_{T}\right)\right\}\;g\left(x_{T}\right)dx_{T}, \end{array}$$

$$\begin{array}{c} \eta_{x_{T}}=-[\begin{array}{c} \ln\{\beta {\left(\frac{\theta }{\alpha }\right)}^{\beta }\}\int_{\tau }^{\infty }g\left(x_{T}\right)dx_{T}+\int_{\tau }^{\infty }\left(\beta -1\right)\ln\left(x_{T}-\tau \right)g\left(x\right)dx_{T}\\ -{\left(\frac{\theta }{\alpha }\right)}^{\beta }\int_{\tau }^{\infty }{\left(x_{T}-\tau \right)}^{\beta }g\left(x_{T}\right)dx_{T} \end{array}], \end{array}$$

$$\begin{array}{c} \eta_{x_{T}}=[\begin{array}{c} {\left(\frac{\theta }{\alpha }\right)}^{\beta }\int_{\tau }^{\infty }{\left(x_{T}-\tau \right)}^{\beta }g\left(x_{T}\right)dx_{T}-\left(\beta -1\right)\int_{\tau }^{\infty }\ln\left(x_{T}-a\right)g\left(x\right)dx_{T}\\ -\ln\{\beta {\left(\frac{\theta }{\alpha }\right)}^{\beta }\}\int_{\tau }^{\infty }g\left(x_{T}\right)dx_{T} \end{array}], \end{array}$$

$$\begin{array}{c} \eta_{x_{T}}={\left(\frac{\theta }{\alpha }\right)}^{\beta }I_{1}-\left(\beta -1\right)I_{2}-\ln\{\beta {\left(\frac{\theta }{\alpha }\right)}^{\beta }\}. \end{array}$$ (25)

$$\begin{array}{c} I_{1}=\beta {\left(\frac{\theta }{\alpha }\right)}^{\beta }\int_{a}^{\infty }{\left(x_{T}-a\right)}^{2\beta -1}e^{-{\left\{\frac{\theta (x_{T}-a)}{\alpha }\right\}}^{\beta }}dx_{T}, \end{array}$$

let

$$\begin{array}{c} {\left\{\frac{\theta (x_{T}-a)}{\alpha }\right\}}^{\beta }=u, \end{array}$$

$$\begin{array}{c} I_{1}={\left(\frac{\theta }{\alpha }\right)}^{-\beta }\int_{0}^{\infty }ue^{-u}du, \end{array}$$

$$\begin{array}{c} I_{1}={\left(\frac{\alpha }{\theta }\right)}^{\beta }, \end{array}$$

$$\begin{array}{c} I_{2}=\int_{a}^{\infty }\ln\left(x_{T}-a\right)g\left(x_{T}\right)dx_{T}, \end{array}$$

$$\begin{array}{c} I_{2}=\beta {\left(\frac{\theta }{\alpha }\right)}^{\beta }\int_{a}^{\infty }\{\ln(x_{T}-a)\}{\left(x_{T}-a\right)}^{\beta -1}e^{-{\left(\theta /\alpha \;\right)}^{\beta }{\left(x_{T}-a\right)}^{\beta }}dx_{T}, \end{array}$$

$$\begin{array}{c} {\left(x_{T}-a\right)}^{\beta }=u, \end{array}$$

$$\begin{array}{c} \beta {\left(\frac{\theta }{\alpha }\right)}^{\beta }\int_{a}^{\infty }\{\ln(x-\tau )\}{\left(x-\tau \right)}^{\beta -1}e^{-{\left(\theta /\alpha \;\right)}^{\beta }{\left(x-\tau \right)}^{\beta }}dx=\frac{1}{\beta }{\left(\frac{\theta }{\alpha }\right)}^{\beta }\int_{0}^{\infty }\ln ue^{-{\left(\theta /\alpha \;\right)}^{\beta }u}du, \end{array}$$

$$\begin{array}{c} I_{2}=\frac{1}{\beta }{\left(\frac{\theta }{\alpha }\right)}^{\beta }\int_{0}^{\infty }\ln ue^{-{\left(\theta /\alpha \;\right)}^{\beta }u}du. \end{array}$$

To solve the above integral, we use the following combinations of logarithms and exponential given by [30] (Jeffrey and Zwillinger. 2007, 7th edition, Eq. (4.331.1), p. 571).

∫0∞lnue-μudu=-1μ(C+lnμ)[Reμ>0],

$$\begin{array}{c} I_{2}=-\frac{1}{\beta }\{C+\beta \ln(\frac{\theta }{\alpha })\}. \end{array}$$

By using *I*_1_ and *I*_2_, Eq (25) becomes

$$\begin{array}{c} \eta_{x_{T}}=1+\frac{\left(\beta -1\right)}{\beta }\{C+\beta \ln(\frac{\theta }{\alpha })\}-\ln\{\beta {\left(\frac{\theta }{\alpha }\right)}^{\beta }\}. \end{array}$$

**Theorem 2.2**. *The r^th^ order statistics f*_*r*;*n*_ *(x) of a random sample of size n for the W-TEXPD distribution is given by*

$$\begin{array}{c} f_{r;n}\left(x\right)=\beta {\left(\frac{\theta }{\alpha }\right)}^{\beta }{\left(x-\tau \right)}^{\beta -1}{\left\{F(x)\right\}}^{r-1}{\left\{1-F(x)\right\}}^{n-r+1}. \end{array}$$

*Proof*. By definition

$$\begin{array}{c} f_{r;n}\left(x\right)=C_{r;n}g\left(x\right){\left\{G(x)\right\}}^{r-1}{\left\{1-G(x)\right\}}^{n-r}, \end{array}$$

or

$$\begin{array}{c} f_{r;n}\left(x\right)=C_{r;n}f\left(x\right){\left\{F(x)\right\}}^{r-1}{\left\{1-F(x)\right\}}^{n-r} \end{array}$$ (26)

where

$$\begin{array}{c} f\left(x\right)={\left(\frac{\theta }{\alpha }\right)}^{\beta }\beta {\left(x-a\right)}^{\beta -1}e^{-{\left\{\frac{\theta (x-a)}{\alpha }\right\}}^{\beta }},\\ F\left(x\right)=1-e^{-{\left\{\frac{\theta (x-a)}{\alpha }\right\}}^{\beta }}\\ f_{r;n}\left(x\right)=\beta {\left(\frac{\theta }{\alpha }\right)}^{\beta }{\left(x-\tau \right)}^{\beta -1}{\left\{F(x)\right\}}^{r-1}{\left\{1-F(x)\right\}}^{n-r+1}. \end{array}$$ (27)

## 3 Estimation of model parameters by using Maximum Likelihood (ML) method

In this Section, we estimate the unknown parameters of W-TEXPD by applying maximum likelihood estimation method as defined by [31]. The log-likelihood function of W-TEXPD is given by:

$$\begin{array}{c} \ln L\left(\tau ,\theta ,\alpha ,\beta ;x\right)=\ln\prod_{i=1}^{n}[\beta {\left(\frac{\theta }{\alpha }\right)}^{\beta }{\left(x-\tau \right)}^{\beta -1}e^{-{\left\{\frac{\theta (x-\tau )}{\alpha }\right\}}^{\beta }}],\\ \ln L\left(\tau ,\theta ,\alpha ,\beta ;x\right)=[\begin{array}{c} n\ln\beta +n\beta \ln\theta -n\beta \ln\alpha +\beta \sum_{i=1}^{n}\ln\left(x_{i}-\tau \right)-\sum_{i=1}^{n}\ln\left(x_{i}-\tau \right)\\ -{\left(\frac{\theta }{\alpha }\right)}^{\beta }\sum_{i=1}^{n}{\left(x_{i}-\tau \right)}^{\beta } \end{array}]. \end{array}$$ (28)

Now computing the first partial derivatives of (28) with respect to *τ*, *θ*, *α*, *β* and equating the results to zero, we have

$$\begin{array}{c} \frac{\partial \ln L(\tau ,\theta ,\alpha ,\beta ;x)}{\partial \tau }=Min\left[x_{i}\right],\;i=0,1,2..........,n, \end{array}$$ (29)

$$\begin{array}{c} \frac{\partial \ln L(\tau ,\theta ,\alpha ,\beta ;x)}{\partial \theta }=\frac{n\beta }{\theta }-\frac{\beta \theta^{\beta -1}}{\alpha^{\beta }}\sum_{i=1}^{n}{\left(x_{i}-\tau \right)}^{\beta }=0, \end{array}$$ (30)

$$\begin{array}{c} \frac{\partial \ln L(\tau ,\theta ,\alpha ,\beta ;x)}{\partial \alpha }=\beta \theta^{\beta }\alpha^{-(\beta +1)}\sum_{i=1}^{n}{\left(x_{i}-\tau \right)}^{\beta }-\frac{n\beta }{\alpha }=0, \end{array}$$ (31)

$$\begin{array}{c} \frac{\partial \ln L(\tau ,\theta ,\alpha ,\beta ;x)}{\partial \beta }=[\begin{array}{c} \frac{n}{\beta }+n\ln\theta -n\ln\alpha +\sum_{i=1}^{n}\ln\left(x_{i}-\tau \right)-{\left(\frac{\theta }{\alpha }\right)}^{\beta }\ln(\frac{\theta }{\alpha })\sum_{i=1}^{n}\left(x_{i}-\tau \right){}^{\beta }\\ -{\left(\frac{\theta }{\alpha }\right)}^{\beta }\sum_{i=1}^{n}\left(x_{i}-\tau \right){}^{\beta }\ln\left(x_{i}-\tau \right) \end{array}]=0, \end{array}$$ (32)

respectively. Since the Eqs (30) to (32) are not in closed form, we use a well-known iterative method i.e. Newton Raphson to obtain the approximate ML estimates for the parameters *θ*, *α* and *β*.

### 3.1 Asymptotic confidence bounds

It is observed that ML estimates of the unknown parameters *θ*, *α*, *β* of W-TEXPD are not in closed forms. In this situation, we compute the asymptotic confidence bounds of W-TEXPD based on the asymptotic distribution of the MLE.

The Fisher Information matrix can be used for interval estimation and hypothesis testing. For W-TEXPD, Information matrix is obtained by computing the second partial derivatives of the Eqs (30) to (32) as:

$$\begin{array}{c} I_{n}=(\begin{array}{ccc} I_{\alpha \alpha } & I_{\alpha \beta } & I_{\alpha \theta }\\ I_{\beta \alpha } & I_{\beta \beta } & I_{\beta \theta }\\ I_{\theta \alpha } & I_{\theta \beta } & I_{\theta \theta } \end{array}), \end{array}$$

the entries of Fisher Information matrix of W-TEXPD are:

$$\begin{array}{c} I_{\alpha \alpha }=\frac{\partial^{2}\ln L\left(\tau ,\theta ,\alpha ,\beta ;x\right)}{\partial \alpha^{2}}=\frac{n\beta }{\alpha^{2}}-\frac{\beta \left(\beta +1\right)\theta^{\beta }}{\alpha^{\beta +2}}\sum_{i=1}^{n}{\left(x_{i}-\tau \right)}^{\beta }. \end{array}$$ (33)

$$\begin{array}{c} I_{\beta \beta }=\frac{\partial^{2}\ln L\left(\tau ,\theta ,\alpha ,\beta ;x\right)}{\partial \beta^{2}}=[\begin{array}{c} -\frac{n}{\beta^{2}}-2{\left(\frac{\theta }{\alpha }\right)}^{\beta }\ln(\frac{\theta }{\alpha })\sum_{i=1}^{n}\left(x_{i}-a\right){}^{\beta }\\ -{\left(\frac{\theta }{\alpha }\right)}^{\beta }\ln(\frac{\theta }{\alpha })\sum_{i=1}^{n}\{\ln(x_{i}-\tau )\}\left(x_{i}-\tau \right){}^{\beta }\\ -{\left(\frac{\theta }{\alpha }\right)}^{\beta }\ln{\left(\frac{\theta }{\alpha }\right)}^{\beta }\sum_{i=1}^{n}\{\ln(x_{i}-\tau )\}\left(x_{i}-\tau \right){}^{\beta }\\ -2{\left(\frac{\theta }{\alpha }\right)}^{\beta }\sum_{i=1}^{n}\{\ln(x_{i}-\tau )\}\left(x_{i}-\tau \right){}^{\beta } \end{array}]. \end{array}$$ (34)

$$\begin{array}{c} I_{\theta \theta }=\frac{\partial^{2}\ln L\left(\tau ,\theta ,\alpha ,\beta ;x\right)}{\partial \theta^{2}}=-\frac{n\beta }{\theta^{2}}-\frac{\beta \left(\beta -1\right)\theta^{\beta -2}}{\alpha^{\beta }}\sum_{i=1}^{n}{\left(x_{i}-\tau \right)}^{\beta }. \end{array}$$ (35)

$$\begin{array}{c} I_{\theta \alpha }=\frac{\partial \ln L(\tau ,\theta ,\alpha ,\beta ;x)}{\partial \theta \partial \alpha }=\frac{\beta^{2}\theta^{\beta -1}}{\alpha^{\beta +1}}\sum_{i=1}^{n}{\left(x_{i}-\tau \right)}^{\beta }. \end{array}$$ (36)

$$\begin{array}{c} I_{\theta \beta }=\frac{\partial \ln L(\tau ,\theta ,\alpha ,\beta ;x)}{\partial \theta \partial \beta }=-[\begin{array}{c} \theta^{\beta -1}\alpha^{-\beta }\sum_{i=1}^{n}\left(x_{i}-\tau \right){}^{\beta }+\beta \theta^{\beta -1}\alpha^{-\beta }\ln\theta \sum_{i=1}^{n}\left(x_{i}-\tau \right){}^{\beta }\\ +\beta \theta^{\beta -1}\alpha^{-\beta }\ln\alpha \sum_{i=1}^{n}\left(x_{i}-\tau \right){}^{\beta }\\ +\beta \theta^{\beta -1}\alpha^{-\beta }\sum_{i=1}^{n}\left(x_{i}-\tau \right){}^{\beta }\sum_{i=1}^{n}\{\ln(x_{i}-\tau )\} \end{array}]. \end{array}$$ (37)

$$\begin{array}{c} I_{\alpha \beta }=\frac{\partial \ln L(\tau ,\theta ,\alpha ,\beta ;x)}{\partial \alpha \partial \beta }=-[\begin{array}{c} \theta^{\beta }\alpha^{-(\beta +1)}\sum_{i=1}^{n}\left(x_{i}-\tau \right){}^{\beta }+\beta \theta^{\beta }\alpha^{-(\beta +1)}\ln\theta \sum_{i=1}^{n}\left(x_{i}-\tau \right){}^{\beta }\\ +\beta \theta^{\beta }\alpha^{-(\beta +1)}\ln\alpha \sum_{i=1}^{n}\left(x_{i}-\tau \right){}^{\beta }\\ +\beta \theta^{\beta }\alpha^{-(\beta +1)}\sum_{i=1}^{n}\left(x_{i}-\tau \right){}^{\beta }\sum_{i=1}^{n}l\{n(x_{i}-\tau )\}-\frac{n}{\alpha } \end{array}]=0. \end{array}$$ (38)

The asymptotic confidence intervals are obtained by using either the approximate normal distribution or the approximate log-normal distribution of the ML estimates $\hat{g}=\left(\hat{\alpha },\hat{\beta },\hat{\theta }\right)$. The estimated standard errors of $\hat{\alpha },\hat{\beta }$ and $\hat{\theta }$ are expressed as:

$$\begin{array}{c} \sigma \left(\hat{\alpha },\hat{\beta },\hat{\theta }\right)=\sqrt{\sum_{jj}\;},\;where\;\sum ={\left[I_{n}\right]}^{-1}. \end{array}$$

For instance, the expressions for (1 − *ξ*)100% confidence interval of *α* calculated by using the approximate normal distribution and log-normal distribution are

$$\begin{array}{c} \hat{\alpha }\;\pm \;\delta_{\xi /2}\;\sigma \left(\hat{\alpha }\right). \end{array}$$ (39)

and

$$\begin{array}{c} \hat{\alpha }\;\exp\{\pm \;\delta_{\xi /2}\;\sigma \left(\hat{\alpha }\right)/\hat{\alpha }\}, \end{array}$$

or

$$\begin{array}{c} \hat{\alpha }e^{\delta_{\xi /2\;}\sigma \left(\hat{\alpha }\right)/\hat{\alpha }\;}\leq \alpha \leq \hat{\alpha }e^{\delta_{\xi /2\;}\sigma \left(\hat{\alpha }\right)/\hat{\alpha }\;}, \end{array}$$ (40)

respectively, where *δ*_*ξ*/2_ is the 1 − *δ*_*ξ*/2_ percentile of standard normal distribution. The log-normal approximation works well if the standard error of parameters is greater than half of their point estimate.

## 4 Simulation study

The core feature of probability is randomness and uncertainty. The randomness exists in every field of life. Simulation imitates the realization of a random experiment, so that random values are generated (that are deterministic) by using an appropriate model designed on the basis of random experiment. A simple such model can be a probability distribution that is used to sketch a real mechanism that produces values of some quantity of interest.

Here, we carry out Monte Carlo simulation studies to assess the performance of maximum likelihood estimates (MLEs) using the R programming. The Monte Carlo simulations are run 1000 times and in each replication, random sample of size n is drawn from the W-TEXPD (*α*, *β*, *θ*). The model parameters are estimated by maximum likelihood method.

Table 1 presents the average point estimates of three parameters with standard errors (SEs), bias and mean square errors (MSEs) for the sample sizes 20, 50, 100 and 200. A fixed seed is used to generate such random numbers implying that all results of these studies can always be exactly replicated.

**Table 1:** Average estimated values, corresponding SEs (given in parentheses) bias and MSE of model parameters.

Actual	n	Average Estimate (S.E)			Bias			MSE		
		$\hat{\alpha }$	$\hat{\beta }$	$\hat{\theta }$	$\hat{\alpha }$	$\hat{\beta }$	$\hat{\theta }$	$\hat{\alpha }$	$\hat{\beta }$	$\hat{\theta }$
*α* = **0.1** *β* = **0.5** *θ* = **2.0**	**20**	0.08583 (0.00021)	0.66002 (0.00208)	1.99978 (0.00003)	-0.01417	0.16001	-0.00001	0.00007	0.02991	0.00001
	**50**	0.09253 (0.00005)	0.72130 (0.00116)	1.99985 (0.00001)	-0.00747	0.22130	-0.00014	0.00005	0.05033	0.00000
	**100**	0.09603 (0.00002)	0.73558 (0.00103)	1.99999 (0.00004)	-0.00397	0.23558	-0.00007	0.00001	0.05655	0.00001
	**200**	0.09793 (0.00001)	0.74491 (0.00110)	1.99991 (0.00001)	-0.00207	0.24492	-0.00004	0.00004	0.06119	0.00001
*α* = **0.1** *β* = **1.0** *θ* = **2.0**	**20**	0.09378 (0.00034)	1.03364 (0.00171)	2.00001 (0.00003)	-0.00622	0.03364	0.00001	0.00016	0.00404	0.00001
	**50**	0.09842 (0.00011)	1.02537 (0.00159)	2.00006 (0.00009)	-0.00158	0.02537	0.00006	0.00001	0.00317	0.00000
	**100**	0.09942 (0.00003)	1.02011 (0.00125)	2.00000 (0.00002)	-0.00057	0.02011	0.00000	0.00001	0.00196	0.00000
	**200**	0.09979 (0.00001)	1.01700 (0.00102)	2.00000 (0.00000)	-0.00002	0. 01700	0.00000	0.00000	0.00133	0.00000
*α* = **0.1** *β* = **1.0** *θ* = **5.0**	**20**	0.09399 (0.00032)	1.03973 (0.00196)	5.00016 (0.00001)	-0.00060	0.03973	0.00016	0.00014	0.00542	0.00001
	**50**	0.09857 (0.00001)	1.02870 (0.00171)	5.00001 (0.00000)	-0.00142	0.02871	0.00001	0.00001	0.00377	0.00000
	**100**	0.09950 (0.00003)	1.02194 (0.00130)	5.00000 (0.00000)	-0.00005	0.02194	0.00000	0.00001	0.00218	0.00000
	**200**	0.09980 (0.00001)	1.01827 (0.00105)	5.00000 (0.00000)	-0.00002	0.01827	0.00000	0.00000	0.00144	0.00000
*α* = **2.5** *β* = **1.0** *θ* = **2.0**	**20**	2.50850 (0.00301)	1.00439 (0.00096)	2.00047 (0.00031)	0.00850	0.00439	0.00047	0.00915	0.00095	0.00010
	**50**	2.50775 (0.00126)	1.01231 (0.00132)	2.00001 (0.00000)	0.00775	0.01231	0.00001	0.00166	0.00189	0.00000
	**100**	2.50491 (0.00079)	1.01451 (0.00130)	2.00000 (0.00000)	0.00491	0.01451	0.00000	0.00065	0.00191	0.00000
	**200**	2.50192 (0.00015)	1.01335 (0.00097)	2.00000 (0.00000)	0.00192	0.01335	0.00000	0.00002	0.00113	0.00000

The assessment is based on a simulation study by applying following steps:

1. Generate one thousand samples of size n each using Eq (14).
2. Compute the MLEs for the one thousand samples, say ($\hat{\alpha },\hat{\beta },\hat{\theta }$) for i = 1,2,3,…..,1000.
3. Compute the SEs of the MLEs for the one thousand samples.
4. Compute the biases and mean square errors by using
  $$\begin{array}{c} bias=\frac{1}{1000}\sum_{i=1}^{1000}{\hat{\delta }}_{i}-\delta , \end{array}$$
  and
  $$\begin{array}{c} MSE=\frac{1}{1000}{\sum_{i=1}^{1000}\hat{\sigma_{\alpha }^{2}}+\{bias({\hat{\delta }}_{i})\}}^{2} \end{array}$$

respectively.

Table 1 shows that biases and MSEs vary with respect to n. The biases and MSEs for each parameter approaches to zero as sample size increases.

## 5 Real life application

### 5.1 Application 1: Aeronautical engineering

To demonstrate the strength of W-TEXPD, we use Aeronautical Engineering data set to show that the proposed distribution can be a better model than the base line distributions i.e. Weibull, truncated Exponential (TEXPD), Gamma and Exponential distributions. We re-analyse the data extracted from [32] to illustrate our proposed model. The data given below are the failure times of air conditioning system in an airplane. [33] used Exponentiated Exponential distribution to model the same data and estimate the parameter as well. The data set of 31 observations is recorded as: 23, 261, 87, 7, 120, 14, 62, 47, 225, 71, 246, 21, 42, 20, 5, 12, 120, 11, 3, 14, 71, 11, 14, 11, 16, 90, 1, 16, 52 and 95.

Table 2 reveals certain descriptive statistics regarding set of observations under study which tells that the data set is positive skewed and heavy tailed towards right.

**Table 2:** Descriptive statistics of failure times of 31 air conditioners of airplane.

Min.	Q1	Q2	Mean	Q3	Max.	S.D.	Skewness	Kurtosis
1.00	11.50	21.00	58.03	79.00	261.00	71.21	1.74	5.14

Table 3 provides the estimated values along with standard errors of unknown parameters of W-TEXPD, Weibull, Gamma, Exponential and Truncated Exponential (TEXPD) distributions by using ML method. The negative log-likelihood, Akaike information criterion (AIC) and Bayesian information criterion (BIC) are computed to compare the models. A distribution with the highest negative log-likelihood value and the smallest AIC and BIC values indicates the better model contrary to other fitted distributions. The values in Table 3 verdict that the proposed model has the highest value of negative log-likelihood and the lowest values of AIC and BIC supporting the new suggested distribution.

**Table 3:** Negative log-likelihood values ($\hat{l}$), MLEs of model parameters, the corresponding SEs (given in parentheses) and the statistics AIC and BIC of failure times of 31 air conditioning system of airplane.

Model	Estimates					Statistic	
	$\hat{l}$	$\hat{a}$	$\hat{\alpha }$	$\hat{\beta }$	$\hat{\theta }$	*AIC*	*BIC*
**W-TEXPD**	**-153.67**	**1.00**	**0.145** (0.070)	**0.738** (0.105)	**0.003** (0.001)	**313.35**	**317.65**
Weibull	-156.10	—	52.883 (11.853)	0.848 (0.116)	—	316.21	319.079
Gamma	-156.39	—	0.014 (0.004)	0.807 (0.176)	—	316.77	319.64
Exponential	-156.89	—	0.017 (0.003)	—	—	315.78	317.22
TEXPD	-156.353	1.00	0.018 (0.003)	—	—	314.71	316.32

Table 4 provides the values of different test statistics which are used to analyse the goodness of fit for the distributions. The distribution having the smallest value of test statistics fits the best. It is obvious from values in the Table 4 that the W-TEXPD distribution leads to a better fit than the Weibull, Gamma, Exponential and TEXPD distributions.

**Table 4:** Goodness of fit statistic of failure times of 31 air conditioners of airplane.

Model	W-TEXPD	Weibull	Gamma	Exponential	TEXPD
K-Smirnov	**0.140**	0.159	0.176	0.221	0.228
C-Von	**0.091**	0.118	0.143	0.253	0.284
A-Darling	**0.633**	0.659	0.783	1.359	1.734

**Fig 3:**
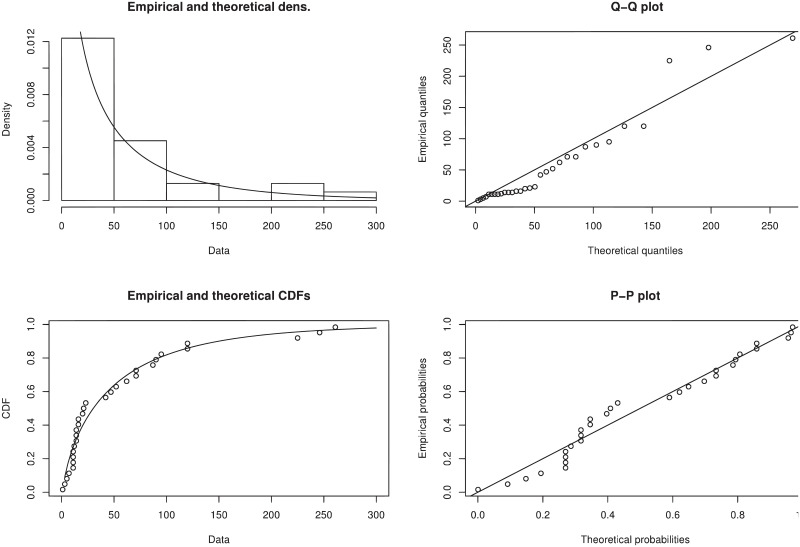
The fitted pdf of W-TEXPD on the histogram of failure times of 31 air conditioning system of airplane along with their cdf, Q-Q and probability plots.

We graphically study the efficacy of new proposed distribution W-TEXPD by sketching pdf, Q-Q, cdf and P-P plots for the above data set to check the goodness of fit. From Fig 3, it is evident from pdf plot that theoretical/predicted probabilities are closer to the empirical histogram which highlights that W-TEXPD better fits the data set. The corresponding cdf, Q-Q and P-P plots suggest the same results as well. It is also observed that the W-TEXPD follows the diagonal line more closely than the empirical line.

### 5.2 Application 2: Electrical engineering

In this section, we use a real data set to show that the W-TEXPD distribution can be a better model than Weibull, truncated Exponential (TEXPD), Gamma and Exponential distributions. The following data set is taken from [7] which represents the failure times of 50 components (per 1000h). [34] use McDonald modified Weibull distribution to model the same data set. The data set of 50 observations is recorded as: 0.036, 0.058, 0.061, 0.074, 0.078, 0.086, 0.102, 0.103, 0.114, 0.116, 0.148, 0.183, 0.192, 0.254, 0.262, 0.379, 0.381, 0.538, 0.570, 0.574, 0.590, 0.618, 0.645, 0.961, 1.228, 1.600, 2.006, 2.054, 2.804, 3.058, 3.076, 3.147, 3.625, 3.704, 3.931, 4.073, 4.393, 4.534, 4.893, 6.274, 6.816, 7.896, 7.904, 8.022, 9.337, 10.940, 11.020, 13.880, 14.730 and 15.080.

The descriptive statistics in Table 5 connotes that the data are highly spread, skewed and long right tailed.

**Table 5:** Descriptive statistics of failure times of 50 components (per 1000 hours).

Min.	Q1	Q2	Mean	Q3	Max.	S.D.	Skewness	Kurtosis
0.036	0.207	1.414	3.329	4.499	15.080	4.184	1.423	4.092

Table 6 provides parameter estimates of the fitted distributions along with ($\hat{l}$), AIC and BIC values. The above table shows that the suggested model leads to better fit than the baseline distributions i.e. Weibull & TEXPD, Gamma and Exponential distributions for describing the certain data.

**Table 6:** Negative log-likelihood values ($\hat{\ell }$), MLEs of model parameters, the corresponding SEs (given in parentheses) and the statistics AIC and BIC of failure time of 50 components (per 1000 hours).

Model	Estimates					Statistic	
	$\hat{l}$	$\hat{a}$	$\hat{\alpha }$	$\hat{\beta }$	$\hat{\theta }$	*AIC*	*BIC*
**W-TEXPD**	**-95.86**	**0.036**	**-0.279** (4.755)	**0.583** (0.067)	**-0.124** (2.111)	**197.73**	**203.46**
Weibull	-102.07	—	2.514 (0.568)	0.661 (0.074)	—	208.14	211.97
Gamma	-102.18	—	0.163 (0.042)	0.545 (0.091)	—	208.37	212.19
Exponential	-110.13	—	0.300 (0.042)	—	—	222.25	224.17
TEXPD	-109.58	0.036	0.304 (0.042)	—	—	221.17	223.08

The smallest values of goodness of fit statistic, i.e. K-S, C-Von and A-Darling for W-TEXPD in Table 7 prove that the proposed model fits the given data best among the other models.

**Table 7:** Goodness of fit statistic of failure time of 50 components (per 1000 hours).

Model	W-TEXPD	Weibull	Gamma	Exponential	TEXPD
K-Smirnov	**0.140**	0.159	0.176	0.221	0.228
C-Von	**0.091**	0.118	0.143	0.253	0.284
A-Darling	**0.633**	0.659	0.783	1.359	1.734

**Fig 4:**
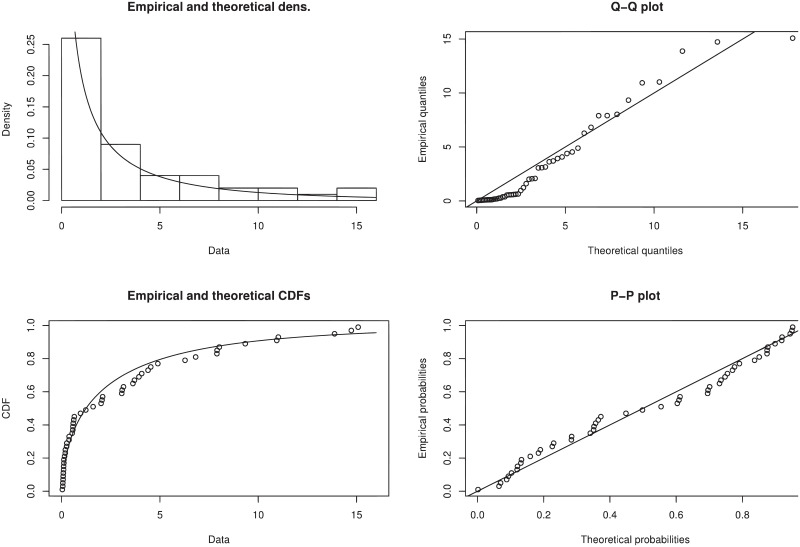
The fitted pdf of W-TEXPD on the histogram of failure times of 50 components (per 1000 hours) along with their cdf, Q-Q and probability plots.

We graphically studied the performance of W-TEXPD by sketching pdf, Q-Q, cdf and P-P plots. We can observe in Fig 4 that the closer empirical and theoretical lines show that the W-TEXPD better fits the above data.

### 5.3 Application 3: Mechanical engineering

To demonstrate the strength of W-TEXPD, we fit our suggested model on the data extracted from [35]. The following observations are the numbers of revolutions (in millions) before failure of 23 ball bearings in a life testing experiment. [28, 36] use the Gamma-Weibull and extended generalized Gamma distributions to model the same data respectively. The data set of 23 observations is: 17.88, 28.92, 33.00, 41.52, 42.12, 45.6, 48.8, 51.84, 51.96, 54.12, 55.56, 67.8, 68.64, 68.64, 68.88, 84.12, 93.12, 98.64, 105.12, 105.84, 127.92, 128.04, 173.4.

Table 8 reveals certain descriptive statistics of the under study data suggesting that the data are slightly skewed and right tailed.

**Table 8:** Descriptive statistics of ball bearing data.

Min.	Q1	Q2	Mean	Q3	Max.	S.D.	Skewness	Kurtosis
17.88	47.20	67.80	72.24	95.88	173.40	37.48	0.94	3.49

Table 9 provides parameter estimates. The larger value of ($\hat{l}$) and the smaller values of AIC and BIC reflect a better model. In this aspect, it is evident from the statistics that the suggested model provids a better fit than the baseline distributions i.e. Weibull & TEXPD, Gamma and Exponential distributions for the certain data.

**Table 9:** Negative log-likelihood values, MLEs of the model parameters, the corresponding SEs (in parentheses) along with AIC and BIC values.

Model	Estimates					Statistics	
	$\hat{l}$	$\hat{a}$	$\hat{\alpha }$	$\hat{\beta }$	$\hat{\theta }$	*AIC*	*BIC*
**W-TEXPD**	**-112.91**	**16.25**	**1.18** (2.56)	**1.52** (0.25)	**0.02** (0.04)	**231.81**	**235.22**
Weibull	-113.69	—	2.10 (0.33)	81.90 (8.60)	—	231.38	233.64
Gamma	-113.03	—	4.03 (1.14)	0.06 (0.02)	—	230.05	232.32
Exponential	-121.44	—	0.01 (0.003)	—	—	244.88	246.01
TEXPD	-115.58	—	0.02 (0.004)	—	—	233.15	234.29

Table 10 gives numerical values of goodness of fit tests. We can make an upshot from these statistical values that suggested model better fits to the above data.

**Table 10:** Goodness of fit statistic.

Model	W-TEXPD	Weibull	Gamma	Exponential	TEXPD
**K-Smirnov**	0.109	0.151	0.123	0.307	0.233
**C-Von**	0.034	0.582	0.039	0.537	0.240
**A-Darling**	0.240	0.329	0.216	2.814	1.272

**Fig 5:**
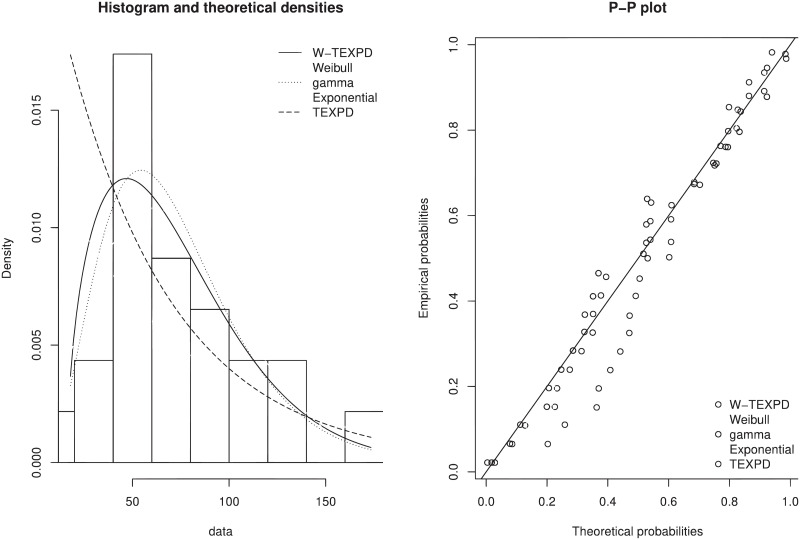
Estimated pdf and P-P plot of W-TEXPD, Weibull, Gamma, Exponential and TEXPD distributions.

The histogram that is superimposed by the empirical pdf in Fig 5 also suggest that W-TEXPD better fits the data. Similarly P-P plot also support the proposed model and gives evidence that it is a pliable probability model for such type of data.

### 5.4 Application 4: Bio-chemical engineering

This example consist of vinyl chloride data which has been taken from the clean up-gradient monitoring wells in *μ*mg/L. [37] utilized following data set to estimate the upper confidence level (UCL) for the mean Vinyl chloride to facilitate the researchers to compare it with the average obtained from down-gradient monitoring well. Similarly, [38] also model the same data by using a New Extended Burr XII distribution. The observations of the Vinyl Chloride data are: 0.2, 2.0, 1.2, 1.3, 0.6, 0.5, 2.4, 0.5, 1.1, 8.0, 0.8, 0.4, 0.6, 0.9, 0.4, 6.8, 1.2, 0.5, 5.3, 3.2, 2.7, 2.9, 2.3, 1.0, 0.2, 2.5, 0.1, 0.1, 1.8, 0.9, 2.0, 4.0, 0.4, 5.1.

Table 11 displays certain descriptive statistics of the under study data reflecting that the data set is skewed and right tailed.

**Table 11:** Descriptive statistics of Vinyl chloride data (*μ*g/L).

Min.	Q1	Q2	Mean	Q3	Max.	S.D.	Skewness	Kurtosis
0.10	0.50	1.15	1.88	2.47	8.00	1.95	1.60	5.01

The statistics in Table 12 display that the suggested model W-TEXPD better explains the vinyl chloride data than the classical probability distributions.

**Table 12:** Negative log-likelihood values, MLEs of model parameters, the corresponding SEs (in parentheses) along with the AIC and BIC values.

Model	Estimates					Statistic	
	$\hat{l}$	$\hat{a}$	$\hat{\alpha }$	$\hat{\beta }$	$\hat{\theta }$	*AIC*	*BIC*
**W-TEXPD**	**-53.19**	**0.09**	**0.121** (0.93)	**0.86** (0.12)	**0.07** (0.56)	**112.38**	**116.96**
Weibull	-55.45	—	1.01 (0.13)	1.88 (0.34)	—	114.90	117.95
Gamma	-55.41	—	1.06 (0.22)	0.56 (0.15)	—	114.82	117.88
Exponential	-55.45	—	0.53 (0.09)	—	—	112.90	114.43
TEXPD	-53.77	—	0.56 (0.09)	—	—	109.53	111.06

Table 13 shows the values of goodness of fit test statistics. It is oberved that W-TEXPD has the smallest values which establish that this model fits the best among the rest.

**Table 13:** Goodness of fit statistic.

Model	W-TEXPD	Weibull	Gamma	Exponential	TEXPD
**K-Smirnov**	0.088	0.092	0.097	0.089	0.105
**C-Von**	0.025	0.043	0.051	0.041	0.061
**A-Darling**	0.220	0.282	0.313	0.272	0.486

**Fig 6:**
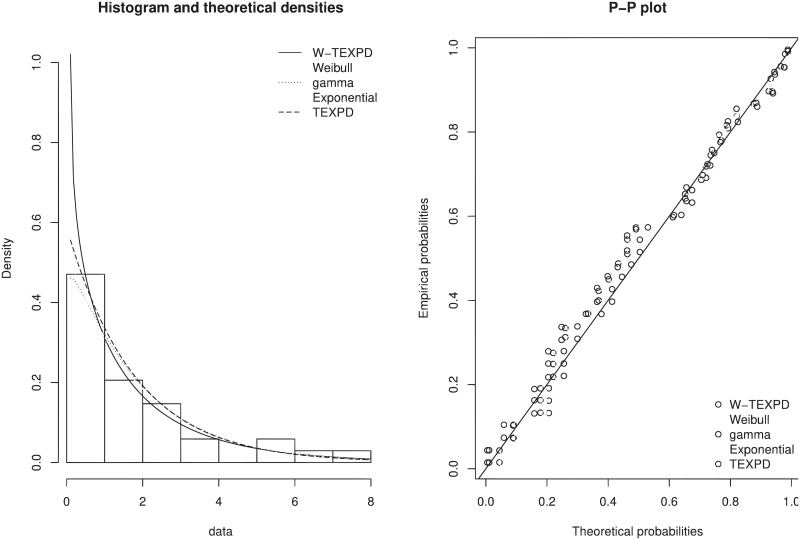
Estimated pdf and P-P plot of W-TEXPD, Weibull, Gamma, Exponential and TEXPD models.

One can clearly observe from histogram and P-P plots of W-TEXPD and the other models given in Fig 6 that the proposed distribution provides the best fit among the competitive models for the Vinyl chloride data.

### 5.5 Application 5: Environmental sciences

The data is taken from a book “Loss Distributions” [39]. In 1977 the following 40 losses, due to wind-related catastrophes, were recorded to the nearest $ 1,000,000. These data include only those losses of 2,000,000 or more; and, for convenience, they have been ordered and recorded in millions. [40] fits Alpha-Power Pareto distribution and [41] fits Lomax exponential model on the same data. The observations in the data set are: 2, 2, 2, 2, 2, 2, 2, 2, 2, 2, 2, 2, 3, 3, 3, 4, 4, 4, 5, 5, 5, 6, 6, 6, 6, 8, 8, 9, 15, 17, 22, 23, 24, 24, 25, 27, 32 and 43.

Table 14 shows that there is a large variation in the data. Furthermore, observations under study are positively skewed and flat towards right.

**Table 14:** Descriptive statistics of 40 losses due to wind-related catastrophes.

Min.	Q1	Q2	Mean	Q3	Max.	S.D.	Skewness	Kurtosis
2.00	2.00	5.00	9.50	13.50	43.00	10.433	1.503	4.369

Table 15 provides the estimated values along with standard errors of unknown parameters by using ML method for W-TEXPD and rest of the fitted models. The negative log-likelihood, Akaike information criterion (AIC) and Bayesian information criterion (BIC) are computed to compare the models. The values in Table 15 highlight that the proposed model is statistically better than Weibull, Gamma, Exponential and Truncated Exponential (TEXPD) distributions.

**Table 15:** Negative log-likelihood values ($\hat{\ell }$), MLEs of model parameters, the corresponding SEs (given in parentheses)along with the AIC and BIC values for 40 losses due to wind-related catastrophes.

Model	Estimates					Statistic	
	$\hat{l}$	$\hat{a}$	$\hat{\alpha }$	$\hat{\beta }$	$\hat{\theta }$	*AIC*	*BIC*
**W-TEXPD**	**-73.96**	**2.00**	**-0.024** (0.025)	**0.359** (0.049)	**-0.008** (0.077)	**153.92**	**158.83**
Weibull	-123.55	—	9.507 (1.637)	1.001 (0.121)	—	251.10	254.37
Gamma	-123.42	—	0.116 (0.029)	1.108 (0.225)	—	250.85	254.12
Exponential	-123.54	—	0.105 (0.017)	—	—	249.10	250.74
TEXPD	-114.57	2.00	0.133 (0.021)	—	—	231.15	232.79

Table 16 provides the values of different test statistics which are used to analyze the goodness of fit of the distributions. The distribution having the smallest value of test statistics fits the best. It is obvious from the values in the Table 16 that the W-TEXPD distribution gives a better fit than the Weibull, Gamma, Exponential and TEXPD distributions.

**Table 16:** Goodness of fit statistic of 40 losses due to wind-related catastrophes.

Model	W-TEXPD	Weibull	Gamma	Exponential	TEXPD
K-Smirnov	**0.224**	0.190	0.205	0.189	0.315
C-Von	**0.422**	0.343	0.398	0.341	1.041
A-Darling	**3.029**	2.055	2.313	2.049	2.165

**Fig 7:**
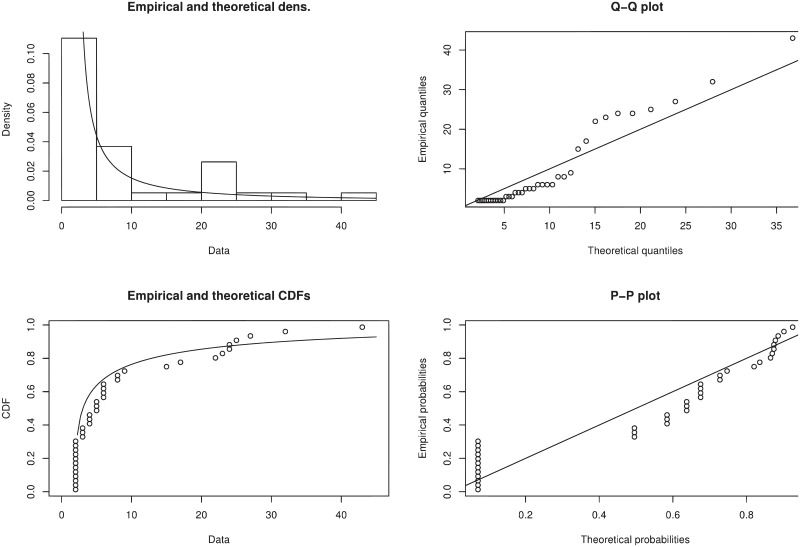
The fitted pdf of W-TEXPD on the histogram of 40 losses due to wind-related catastrophes along with their cdf, Q-Q and probability plots.

The graphical study reveals the performance of the W-TEXPD by sketching pdf, Q-Q, cdf and P-P plots in terms of the goodness of fit. It is evident from Fig 7 that the observed probabilities plotted against the predicted probabilities are closer and follow the diagonal line. Hence, it is concluded that W-TEXPD is the best choice for modeling the above data.

## 6 Concluding remarks

In this research paper, we introduce a new four parameter left truncated distribution called Weibull-Truncated Exponential distribution (W-TEXPD) by employing a new generator. The objective of the present research is to provide a trucated model for finite data. Besides, the additional scale parameter shows a significant impact on the shape of the distribution. A number of distributions are observed as the special cases of the proposed distribution.

It is demonstrated through real life applications that the truncated distribution can quite effectively be used to model a variety of data sets from different fields. It is also concluded that our proposed model better fits the data comprising extreme and/or scattered values as well as skewed (spread) and heavy tailed (flat curved) data. W-TEXPD is effectively applied in engineering and environmental sciences where such type of truncated data are commonly encountered. In future research, we will make a study to compare these estimators for censored data.
